# Diagnosis of Cholecysto-Colonic Fistula Using Gadoxetic Acid – Magnetic Resonance Cholangiography

**DOI:** 10.5334/jbr-btr.849

**Published:** 2015-09-15

**Authors:** A. B. Mourri, M. Lemort, Y. M. Bensouda, J. L. Engelholm

**Affiliations:** 1 Department of Radiology, Université Libre de Bruxelles (ULB), Brussels, Belgium; 2 Department of Science, American School of Tangier (AST), Tangier, Morocco

**Keywords:** Fistula, cholecystocolonic

## Abstract

We report the demonstration (for the first time to our knowledge) of a cholecysto-colonic fistula using Primovist® enhanced MRCP in a 74-year-old patient. We discuss the advantage of this newly emerged technique over traditional T2-weighted MRCP in this indication.

Enterobiliary fistula is an unusual complication of cholelithiasis. It has also been reported in ulcerative colitis, abdominal trauma, Crohn’s disease, and malignancy of the bowel, the head of the pancreas, and the biliary tract. Cholecysto-duodenal fistulas are the most frequent (75%), and only 10–20% are cholecysto-colonic. The majority of patients with cholecysto-colonic fistulae are females and elderly. The typical presentations include diarrhea (most common), nausea, abdominal pain, weight loss, and dyspeptic symptoms [1]. Malabsorption could result when the fistula alters the normal bile acid circulation. The asymptomatic form is not rare. Pre-operative identification is complex and often doubtful. The definitive diagnosis is typically achieved during surgery.

## Case report

**Figure 1:**
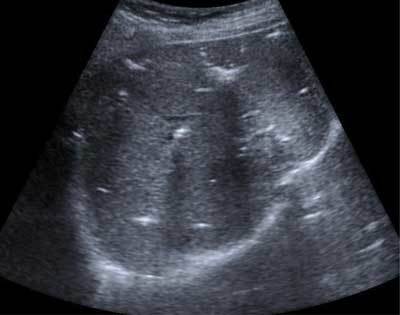
Ultrasound scan, axial view, of liver shows pneumobilia (black arrow).

We discuss the case of a 74-year-old woman treated for breast cancer with bone metastases. Her past medical history also included pulmonary embolism, hypertension, appendectomy, hysterectomy, and aortic valvuloplasty. Annual follow-up ultrasonographic examination of the abdomen showed an unexplained pneumobilia (Fig. 1). She was asymptomatic and during physical examination the patient’s abdomen was soft and she had neither jaundice nor diarrhea. Her blood workup was normal.

**Figure 2:**
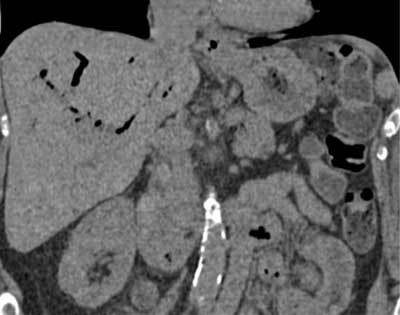
Coronal CT scan shows pneumobilia (white arrow) and common bile duct dilatation with suspicion of lithiasis (black arrow).

**Figure 3:**
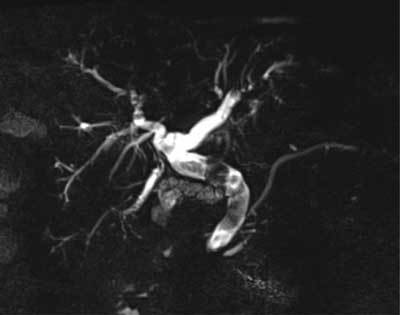
Oblique coronal MRCP image shows a dilated bile duct with numerous large calculi (white arrow).

**Figure 4:**
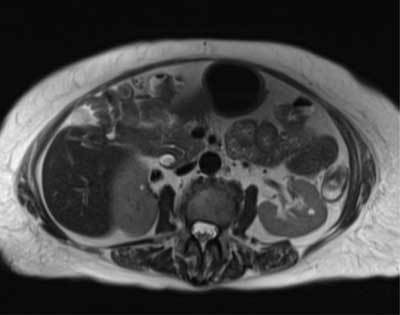
Axial T2-weighted images show a possible communication between the gallbladder and duodenum/colon (white arrow).

**Figure 5:**
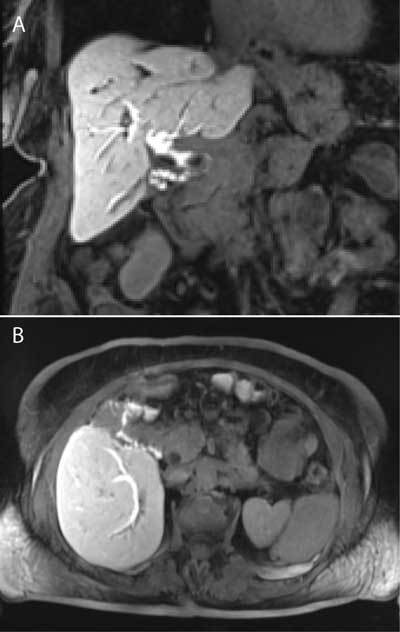
Coronal Fat-suppressed T1-weighted image (A) 90 mm after administration of Primovist® shows the filling of the intrahepatic bile ducts, common hepatic duct and cystic duct (black arrow). Axial Maximum Intensity Projection reconstruction (B) reveals the fistulous tract (white arrow) and presence of contrast in the transverse colon (black arrow).

CT of the abdomen was then performed and confirmed massive pneumobilia and common bile duct dilatation with suspicion of lithiasis (Fig. 2). A T2-weighted sequences magnetic resonance cholangiography (MRCP) displayed a wide bile duct dilatation with numerous large calculi filling the choledocus (Fig. 3). The gallbladder was small and its fundus neighbored the hepatic flexure of the colon and duodenum (Fig. 4). To clarify any potential biliary enteric fistulas, a second MRI was carried out using gadoxetic acid (Gd-EOB-DTPA, Primovist®). The opacification of bile ducts was unusually delayed. The MRI at 90 minutes finally showed the filling of the intrahepatic bile ducts, gallbladder, upper choledocus and opacification of the hepatic flexure of the colon via a fistula (Fig. 5).

Because of her medical history, endoscopic treatment with sphincterotomy and common bile duct stone extraction was favored over surgery.

## Discussion

We report a case of cholecysto-colonic fistula in a female patient, due to bile duct lithiasis. Cholelithiasis is the most common cause of fistula (in more than 90% of the cases) [1]. Cholelithiasis could cause either repeated episodes of acute cholecystitis/cholangitis or asymptomatic chronic calculous cholecystitis [1]. The chronological events that lead to the fistula include gallbladder inflammation, adhesions between intestines and gallbladder, mechanical erosion by gallstones and gangrenous changes of both the gallbladder and the adjacent colon wall.

Enterobiliary fistulae most commonly result from calculus cholecystitis, but can also occur occasionally following carcinoma of the gallbladder where the necrotic tumour perforates into the adjacent duodenum or colon, or both [2]. Due to its proximity, the duodenum is the most common site of intestines involved.

Biliary ileus is defined as a mechanical intestinal obstruction due to a gallstone lodging in the intestinal lumen. The usual means of gallstone entry into the bowel is through a biliary enteric fistula, which complicates few cases of cholelithiasis with associated episodes of cholecystitis. As in calculus cholecystitis enterobiliary fistula and gallbladder carcinoma, the cholecysto-duodenal fistulae are the most common while cholecysto-colic fistulae occur much less frequently. Rarely, cholecystogastric fistulas can also result in biliary ileus.

The fistula remained asymptomatic for a long time, and could only be suspected when an unexplained pneumobilia was found. The pneumobilia showed by abdominal ultrasound or CT, is not specific but may provide presumptive evidence for the presence of a biliary-enteric fistula. Other radiological findings include a shrunken thick-walled gallbladder around gallstones or a small atrophic gallbladder adherent to neighboring organs [3].

Common diagnostic procedures include Barium enema, 99mTc scintigram, CT, MRCP and endoscopic retrograde cholangiopancreatography (ERCP). However, non-visualization of the fistulous tract happens in more or less half of the cases [3].

Contrast-enhanced (CE) MRCP using hepatobiliary contrast agents is a recently emerged technique. Both gadopentetate dimeglumine (Gd-BOPTA, MultiHance®) and Primovist® are incorporated into the hepatocytes by an anionic transport system after the vascular phase. Approximately 3–5 % of the injected dose of MultiHance® and 50 % of Primovist® are excreted in the biliary system [4, 5]. Moreover, Primovist® also provides adequate biliary imaging within a shorter period of time (10–20 min) than MultiHance® [6].

In our case, the opacification of bile ducts was delayed until 90 minutes due to presence of obstructive stones. The conventional T2-weighted MRCP demonstrated possible communication between gall bladder and duodenum/colon (Fig. 4), but Primovist®-MRCP more clearly showed the fistulous tract and presence of contrast in the transverse colon, improving confidence in the fistula diagnosis and depicting its exact anatomical location (Fig. 5). ERCP sphincterotomy, with stone extraction, to reduce biliary pressure was performed. This method has been shown to cause spontaneous healing of fistulas.

In conclusion, Primovist®-enhanced MRCP is a recently emerged examination that provides excellent bile duct analysis. As shown by our case report, it displays biliary anatomy in great detail, increases accuracy in detection of bile duct leak and fistula, and provides additional functional information about bile duct flow.

## Competing Interests

The authors declare that they have no competing interests.
